# A Complex Vibration Analysis of a Drive System Equipped with an Innovative Prototype of a Flexible Torsion Clutch as an Element of Pre-Implementation Testing

**DOI:** 10.3390/s22062183

**Published:** 2022-03-11

**Authors:** Andrzej N. Wieczorek, Łukasz Konieczny, Rafał Burdzik, Grzegorz Wojnar, Krzysztof Filipowicz, Mariusz Kuczaj

**Affiliations:** 1 Department of Mining Mechanization and Robotisation, Faculty of Mining, Safety Engineering and Industrial Automation, Silesian University of Technology, 44-100 Gliwice, Poland; krzysztof.filipowicz@polsl.pl (K.F.); mariusz.kuczaj@polsl.pl (M.K.); 2 Department of Road Transport, Faculty of Transport and Aviation Engineering, Silesian University of Technology, 40-019 Katowice, Poland; lukasz.konieczny@polsl.pl (Ł.K.); rafal.burdzik@polsl.pl (R.B.); grzegorz.wojnar@polsl.pl (G.W.)

**Keywords:** vibrations, flexible torsion clutch, STFT, order analysis, Campbell diagram

## Abstract

The paper presents how an important aspect of introducing new machines, especially in the mining industry, is testing a prototype under laboratory conditions. For this purpose, advanced methods of analyzing the vibrations of a drive system equipped with an innovative prototype of a flexible torsion clutch are presented. The main goal is to present a comprehensive method for analyzing vibration signals in various dimensions of the signal analysis. As a result of this approach, it can be seen how much important information about the tested clutch can be obtained by using various analysis methods in terms of time–frequency distributions or order analysis. To emphasize the differences in the functioning of the tested clutch and the possibility of monitoring these differences on the basis of the observation of residual processes, such as vibrations, the results for the flexible and locked clutch are compared.

## 1. Introduction

The development of new machine concepts requires extensive knowledge and experience of the designer. However, it is absolutely necessary to verify the design assumptions each time and conduct simulation tests [1]. In the case of machine components that directly affect the efficiency of operation and the safety of use, the prototype should be tested further under laboratory conditions as an increase in the TRL (Technology Readiness Level) [2]. The use of TRLs enables consistent and uniform discussions of the technical maturity between different types of technology. During the laboratory tests of the prototype, it is important to obtain the greatest possible amount and the best quality of information about the tested device. Therefore, it is important to design the measurement system properly and develop an appropriate method to analyze the results to extract useful information. In the case of machines and devices operating in difficult conditions, especially if they affect operators and other employees, all tests at the prototyping stage are very important. Therefore, the present paper presents the pre-implementation testing of the prototype of a clutch installed in mining machines, as an element that significantly influences the correct operation of these devices.

Underground extraction is characterized by the high variability of the mining and geological conditions in which it is conducted. Despite ever more effective methods and tools used to identify the factors influencing this process, mining machinery, used in underground mining, works in difficult conditions, which means that these machines should be universal and reliable [3,4]. The reliability and availability of this equipment play critical roles in increasing the efficiency and productivity of a mining operation [5]. Intelligent mining, which is based on mechanized and automated mining methods, recently brought about a revolution in the coal industry. Intelligent mining has three main characteristics: mining machines have the intelligent ability to work by themselves, real-time data can be captured and updated promptly, and machinery can be automatically controlled according to the work conditions [6,7]. An essential element of intelligent mining is the ability to acquire data, i.e., the signals recorded by measuring sensors. Evidently, the signals themselves are only inputs of the processing algorithms and the monitoring and diagnosis systems that determine the decision-making algorithms of process control.

The modern mining industry is characterized by an advanced technical infrastructure equipped with numerous sensors and diagnostic systems. Mining machines allow the mechanization of basic process operations, which make up the drilling process for that type of roadway, that is, rock cutting, loading of the output on the means of transport, and output from the roadway face and roof [8]. A very important element of mining machines is the drive mechanisms responsible for maintaining the operation of mining machines. An example of a device is a scraper conveyor responsible for transporting excavated material from the mining wall. In addition to the obvious phenomena that should be analyzed during the prototyping of this machine related to friction, there are many other phenomena related to the dynamic nature of the work [9,10]. The extremely difficult working conditions of these machines, resulting from the level of dust, working and ambient temperatures, humidity, random loads, including impulse loads that occur very often, but also the very difficult conditions for assembly and maintenance. All of these factors determine that operational forecasting, including repair and maintenance planning, is extremely difficult. Furthermore, the wear characteristics and reliability estimation are subject to considerable uncertainty. This is due to the overlapping of many factors that influence the probability of failure. Evidently, at present, economic aspects are very important at the design stage, including the costs of operating the machines and their energy consumption. Interesting analyses of the example of the influence of the type of belt on the energy consumption rate of the conveyor belt are presented in [11].

Based on the analysis of the problem, the concept of pre-implementation research was developed in relation to the methods of technical diagnostics used in similar mining machines. The developed research concept has the character of additional testing without interfering with the design of the prototype. Therefore, it was decided to use vibration signals. An additional advantage of this approach is the ability to adapt the measurement strategies to monitor and diagnose machines during their normal operation [12,13].

Considering mining machines in theoretical terms, they should be considered as strongly nonlinear systems, and their transmission vibration signals are nonlinear mixtures of different kinds of vibration sources [14]. Therefore, issues related to the diagnosis of the technical condition of this type of machine are very difficult and constitute a research and engineering challenge [12]. In view of the factors previously mentioned, the authors planned a multistage experimental study of mining machine drive elements, during which various physical and electrical quantities of the drive system were registered as symptoms of the operating state. This article presents the results of the analysis from vibration signals of the drive system equipped with an innovative prototype of a flexible torsion clutch.

Vibration analysis is one of the most widely used methods in the detection of mining machine faults, due to its cost-free use and easy installation [12,15,16,17]. The performance of multi-fault detection in machines is extremely difficult, especially if just one source of information is used, e.g., vibration signals. The most fundamental goal of operational diagnostics is to detect the defects in the early stages of the related changes, before the transmission actually becomes damaged. An assessment of the dynamic state of a transmission based on an analysis of vibroacoustic signals is a problem discussed in numerous papers [18,19,20].

Due to the nature of clutch operation, the current trends in measurements and condition monitoring using vibration signals from rotary machines were also analyzed. Machines subjected to time-varying rotational speeds are a difficult challenge for fault diagnosis. Therefore, these issues have become the goal of many researchers [21,22,23,24,25]. In order to analyze the diagnostic signals recorded during the operation of this type of machines, it is often used order tracking, as one of the most effective algorithms that can be used to remove the effects of speed fluctuation and blurring of the spectrum at changing speeds. Therefore, order tracking is also a powerful technique for rotating machine fault diagnosis under variable-speed conditions [26]. The paper [27] presents a review summarizing the recent advances in the development of tacholess speed estimation methods for order tracking and its application to fault diagnosis. Order tracking methods can be applied with or without auxiliary devices, such as tachometers or encoders. If phase markers are recorded or calculated by signal processing, ordering tracking can be implemented with reasonably high accuracy.

Fault detection and classification methods can be divided into two main groups: signal processing-based techniques and model based approaches. Signal processing approaches extract features from the measured signals [28]. The vibration signals are nonlinear and cover results from different sources. Therefore, they should be observed as simultaneous distributions of values in the domains of observation time and frequency of signal components. Therefore, subsequent research assumptions related to the possibilities of the effective analysis of results and separation of information from signals concern methods of analyzing time–frequency representations. The analysis of nonstationary signals requires the transformation of the waveform of the registered changes in values into time–frequency distributions. For this purpose, various mathematical transformation algorithms, such as the Short-Time Fourier Transformation, wavelet transformation or Wigner–Ville distribution and many more, are used for fault detection [14,29,30,31,32].

If the time–frequency representation satisfies the superposition principle, then it is known as Linear Time–Frequency Distribution (LTFR) algorithms [28]. Empirical Mode Decomposition (EMD) is the example of recent advancements developed in LTFR to detect the machinery faults. It is the process to decompose the whole data set into the number of components that satisfies intrinsic mode functions. The paper [33] presents a new method to address the mode mixing problem in EMD-based gearbox multi-fault diagnosis. The proposed method is used for applications in which the vibration signature of the gear fault is strongly masked by background noise and disturbance. If separable fault symptoms can be calculated from diagnostic signals, the risk of undergoing defects in mining machines can be effectively detected to ensure the safe, stable and reliable operation of the machines [33].

The power transmission systems used for mining machines operate in extremely difficult conditions. Transmission torque is often a random variable. It requires the application of high torsional flexibility couplings. A flexible torsional coupling has specific elastic and damping characteristics that determine the operation of the power transmission system. These are the results in the change of traction and stabilization of torsional vibration and load torque [34]. The frequent phenomenon of temporary overloads of the drive transmission system in mining machines causes the compression of elastic elements. The subsequent underloads of the machine (e.g., a belt conveyor or a longwall shearer) cause the return of the elements also dynamically elastic. After the power transmission, in the clutch, the spring-biased nut returns to its initial position on the shaft. If the preload force of this nut is sufficiently high and the transmission system is disconnected, the nut returns to its original position. The torsion angle of the coupling elements at nominal load can be quite large [35]. Electromechanical system solutions are increasingly used in mining machines. Most of the presented research in this field represent an approach as an electromechanical coupling system as uncoupled, and study the vibration characteristics of electric machine and mechanical drive system separately [36]. However, the problems due to mechanical vibration caused by the effect of electromechanical interactions gradually appear and intensify [37].

Based on recent studies and achievements in the field of measurement systems and signal processing in mining machines and devices, authors developed the concept of pre-implementation testing of the drive system equipped with an innovative prototype of a flexible torsion clutch and complex vibration analysis algorithm. Novelties of the research are represented in two areas: as a new approach to extended identification tests at the pre-implementation stage based on non-invasive vibration methods, and as a proposal of a comprehensive method of vibration signal analysis enabling the verification of the clutch operation. A difference in relation to the research carried out to date is also visible in the range and number of measurement points that enable the evaluation of the entire torque transmission mechanism.

## 2. Materials and Methods

The whole research covers a series of many active diagnostic experiments conducted under various laboratory conditions. The topics discussed in this paper include tests in a professional testing station at Patentus, in which comprehensive tests of drive components in mining machines are carried out under fully controlled conditions. The scope of the research conducted here included multibeam measurements of various physical and electrical quantities of the drive system equipped with an innovative prototype of a flexible torsion coupling. To analyze the effectiveness of the clutch operation, loads and moments were recorded, and to analyze the durability, vibroacoustic signals, including vibrations, torsional vibrations, and noise, were recorded.

**Figure 1:**
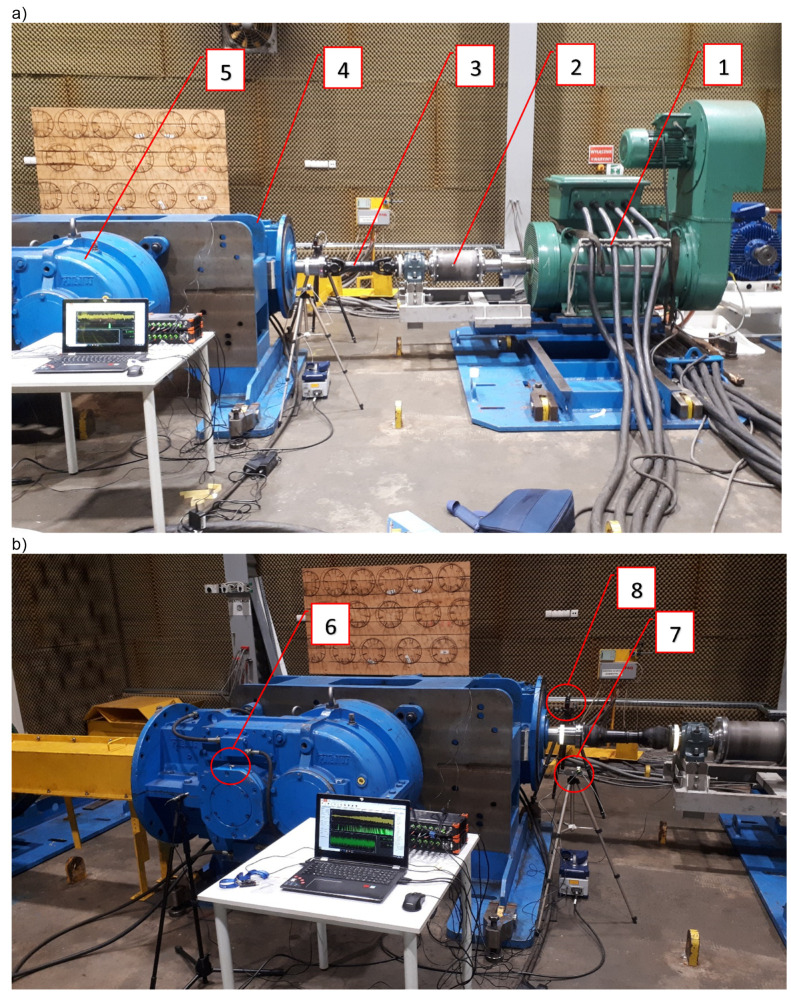
Object of the research: electric motor driving (1), innovative flexible torsion clutch (2), shaft with universal joints (3), set of two multistage gears ((4) and (5)), vibration acceleration converter (6), tachometric sensor (7), and laser vibrometer for non-contact laser measurement of instantaneous angular velocity changes of the shaft by Polytec RLV-5500 (8)—(**a**,**b**) represents different view.

**Figure 2:**
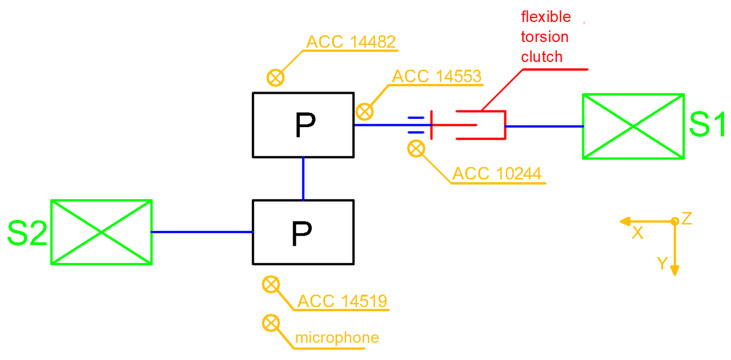
Kinematic diagram of the test stand and points of location of the measurement points.

The main objective of the present research was a comprehensive analysis of the time–frequency vibration signals of the drive system shown in Figure 1, equipped with an innovative clutch, performed at the Patentus company. Comprehensive analysis in terms of technical diagnostics should cover almost all machine load states. This is extremely important when vibration signals are used for diagnostic purposes, because vibration signals are extremely susceptible to any dynamic and kinematic changes. Therefore, the content and scope of the research included, inter alia, a run-up of the test stand to full working speed, i.e., 1480 rpm. During the start-up, no additional load with braking torque was applied, which would be generated, and the braking motor was located behind the multistage gear system (Figure 1, items 4 and 5). Vibration accelerations were recorded with a 3-axis accelerometer mounted on the housing of the bearing housing at the point marked with the number 10244 (Figure 2), directly behind the clutch (looking at the torque transmission from the driving motor).

The object of the research was the drive system shown in Figure 1, consisting of an electric motor driving (1); an innovative flexible torsion clutch (2), which, thanks to a screw mechanism, allows us to obtain high values of torsion angles, for example, during the start-up of the drive system; a shaft with universal joints (3); a set of two multistage toothed gears ((4) and (5)), the first (4) of which worked as a reducer, and the second (5) as a multiplier; and a braking electric motor (Figure 2, item S2). Figure 1b also shows the location of one of the vibration acceleration transducers mounted on one of the bearing housings (6), as well as the tachometric sensor (7) generating a pulse signal once per revolution of the driving shaft, and the location of the laser vibrometer for the non-contact laser measurement of instantaneous angular velocity changes of the company’s shaft Polytec RLV-5500 (8). The distributions of the measurement points are schematically shown in Figure 2.

**Figure 3:**
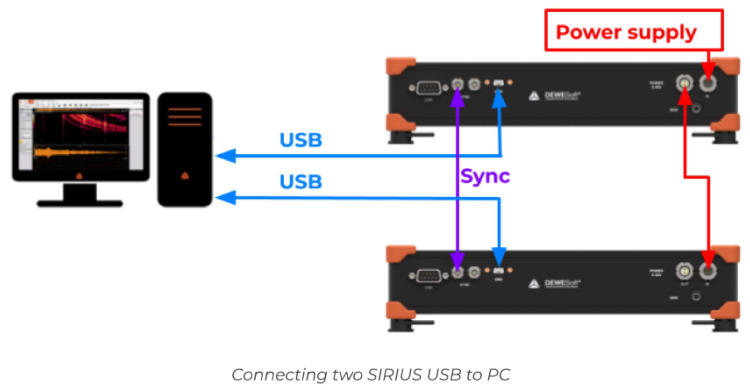
Two chassis were combined and synced to obtain a sixteen-channel solution.

In the case of the conducted research, a hardware and software platform was used, consisting of a Sirius data acquisition card and Dewesoft software. The measurements were made with the use of DEWESOF software supporting SIRIUS data acquisition cards. An 8-channel card with ACC inputs was used in the tests (Figure 3).

This technology enables a full possible measuring range because the signal is measured simultaneously with a high and a low gain. The dynamic range is 160 dB. The Sirius data acquisition device has 8 input channels. The important advantage of this technology is that multiple chassis can be combined and synced together. In the investigation, 2 synchronously connected 8-channel Sirius analyzers (Figure 3) were used.

**Figure 4:**
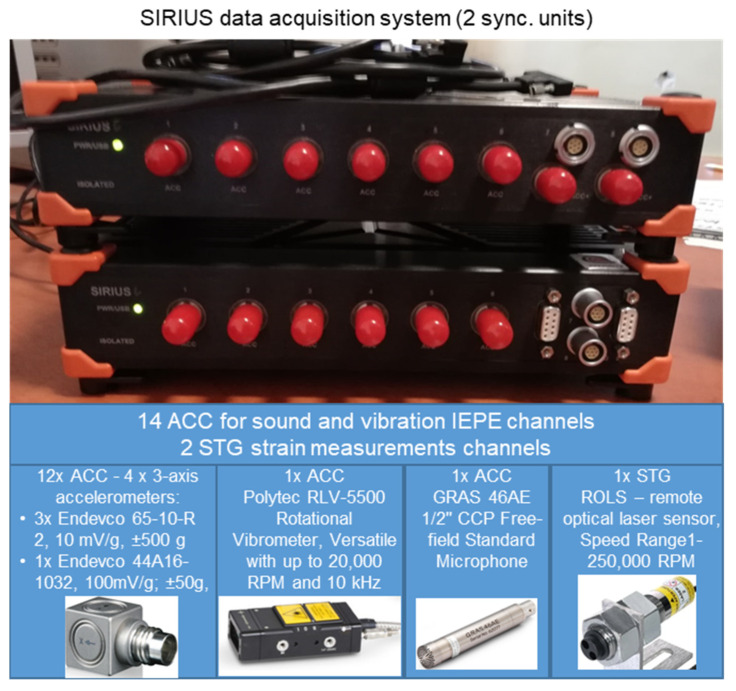
Measurement system with sensor specifications.

The full specification of the measurement system used in the research is shown in Figure 4.

This paper is limited to the analysis of vibrations registered on the bearing housing directly after the clutch, as the first kinematic node transmitting the drive through the innovative flexible torsion coupling (Figure 2, sensor ACC 10244). The selection of the measuring point results from the advantages in terms of the structure of the structural health monitoring system. The vibrations recorded in this place should reveal the damage patterns of the clutch as soon as possible, or constitute a reference set of data on the technical condition and operating parameters of the clutch.

## 3. Method of Analysis

As a result of the studies, transmission vibration signals were recorded. Digital signals were analyzed using MATLAB software.

In order to obtain the greatest possible amount of information from the recorded vibration or acoustic signals, very often time–frequency analyses are used, which, in the case of non-stationary signals, are almost obligatory.

For flexible couplings, the nonlinearity is mostly determined by the mechanical characteristics of the flexible connector. Mechanical characteristics are very important during model testing. A small change in the external load can generate different properties for different solutions [38,39].

The paper [17] Welch test, Short-Term Fourier Transform (STFT), Wigner–Ville Distribution (WVD), and Choi–Williams Distribution (CWD) were used for the detailed examination of vibrations generated by an under-load engine. The results show that the Short-Term Fourier Transform (STFT) is the most efficient method for such a fault detection process. The most important advantage of the STFT in the presented case is the computing time. The average computing times in the STFT, WVD and CWD methods presented in [7] are 0.29, 0.54, and 3.25 s, respectively. Using a simpler algorithm, the STFT is a suitable approach for online and offline knock detection, thus it was chosen for the research presented in this paper. Paper [40] presents the application of the STFT for the analysis of the relative instantaneous angular speed between the two end sections of the flexible coupling during a run-up. The results clearly highlight the phenomenon that determines an early failure of the coupling. The STFT presented in [40] clearly shows that this increase is due to the resonance of the system.

For the vibration acceleration time signals recorded in the *x*, *y*, and *z* directions, STFT analyses were carried out, showing the signal structure in the time and frequency domains at the same time.

**Figure 5:**
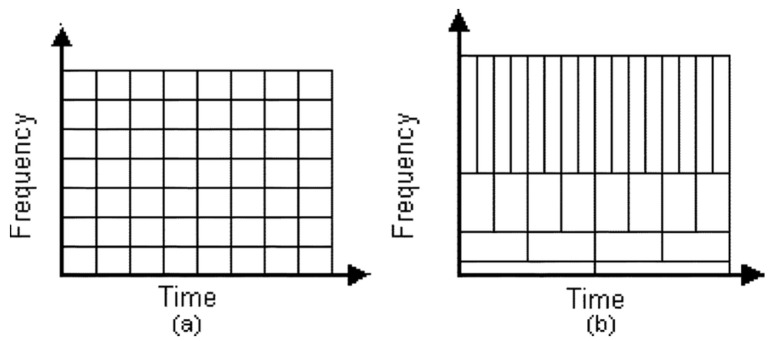
Comparison of the window widths during signal transformation with the STFT (**a**) and WT (**b**).

The Short Time Fourier Transform (STFT), also known as the Window Fourier Transform, is based on a constant and selected width of the analysis window (Figure 5), as opposed to, e.g., the wavelet analysis (WT), which is a compromise of precision in the time domain and frequency of the analysis. The comparison of the composition of the selection of the STFT and WT analysis window is presented in the figure below.

When using time–frequency transformations, it should be realized that the smallest identifiable signal component, called an atom due to further atomicity, corresponds to its size in the two-dimensional time–frequency domain, and to a rectangle whose sides constitute the time and frequency ranges of the basis function analysis. Therefore, the decision to select a signal processing method for the time–frequency representation (TFR) should be preceded by a careful analysis. The minimum acceptable resolution of the transformation (analysis of the window size) should be taken into account, which, in turn, should take into account the separativity of the frequency components and the shortest duration of the symptom. In the case of using, for example, the wavelet analysis, when selecting the type of wavelet, it is worth analyzing the correlation of the shape of the waveform of components. In addition, in the case of real-time monitoring, the time needed for signal processing becomes very important.

The sizes of the windows, through which the fragment of the analyzed signal is obtained, are rigidly imposed and therefore the resolution of the Short-Time Fourier Transform is a compromise between the sensitivity to the features of the time and frequency. With longer window lengths, the low-frequency components are blurred (which results directly from the assumed length of the time window). The increased window length improves the frequency resolution for the low-frequency components but, at the same time, reduces the time domain resolution. Despite some disadvantages, this type of time–frequency analysis is used for the study of nonstationary signals, because of the short computation time and low computational power requirements.

**Figure 6:**
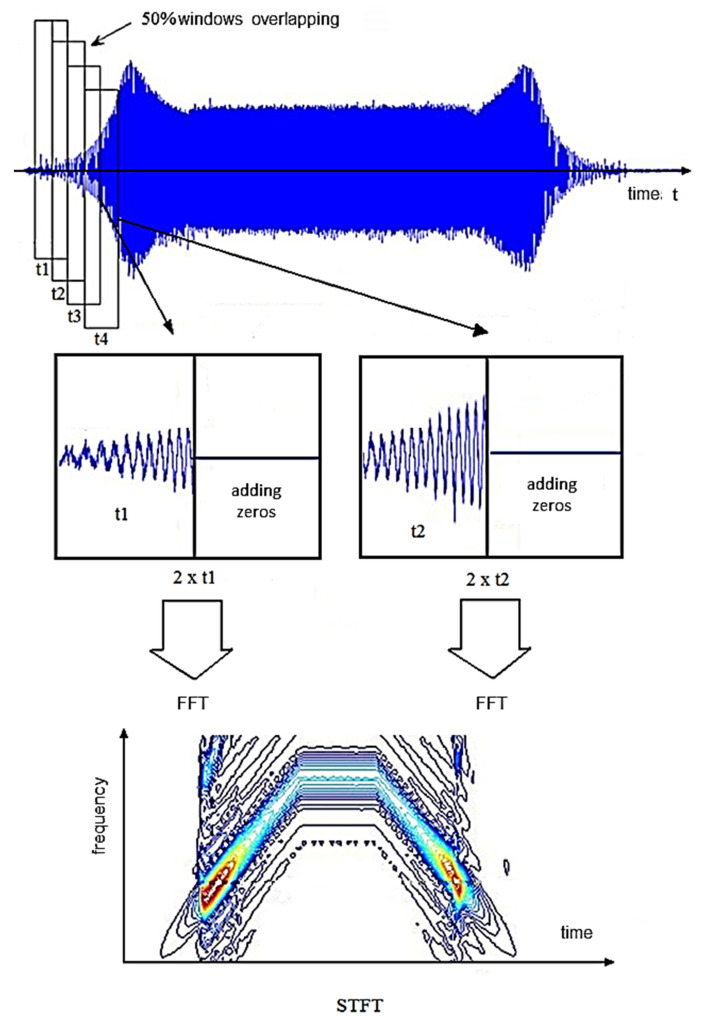
Scheme of signal preprocessing using the STFT with overlapping a window and adding zeros.

Very often, the decisive advantage of choosing the STFT in vibration monitoring systems, which is the short time of signal processing, forces the development of the algorithm to improve the resolution of the analysis. One of the solutions that improves the selectivity of the STFT method is the use of partial window overlap (single samples of signals are used many times in individual FFT analyses). The application of the overlapping window method is shown in Figure 6. This is a simple method for which a single window is used multiple times for a string of inputs.

An important aspect of non-linear dynamics research is the identification of coexisting multiple subharmonic solutions for different initial conditions. The vibration analysis of machines operating under non-stationary operational conditions requires special attention. This refers to the necessity of using order tracking algorithms, together with additional advanced signal processing methods. During all the research, the instantaneous shaft rotation speeds were recorded in a synchronous manner with the use of a laser speed sensor. Then, as part of the vibration signal processing, the order analysis was performed.

**Figure 7:**
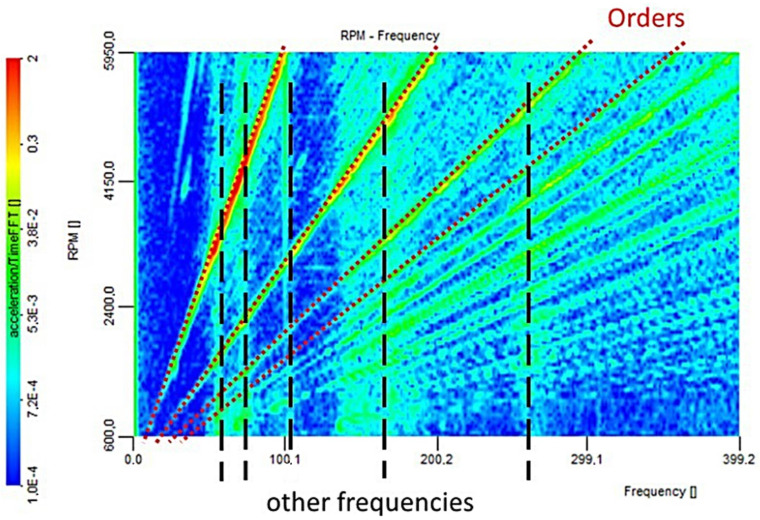
A time–frequency graph with the orders related to the change of speed and other characteristic frequencies marked.

Order analysis is used for rotary machines under conditions of operation with variable rotational speeds. The frequencies generated by the rotating parts of the machine depend on the rotational speed. In the event of velocity changes during the sampling, the signal from one element generates a variable-frequency signal corresponding to the velocity changes, blurring the frequency spectrum. The characteristic bands in the spectrum of different elements may overlap and prevent frequency analysis. In the case of transient processes in the operation of rotary machines, such as in-run changes or changes in the operating speed, the rotational speed is a function of time. In this case, for the recognition of the frequency structure of the tested signal, time–frequency analysis tools should be used. One of these tools is the analysis of orders in the diagnostics of nonstationary vibroacoustic processes, which presents information about the variability of individual harmonics over time (Figure 7).

Individual rotational-speed-dependent harmonics are related to the same function that describes the changes in rotational speed with time. Order analysis enables this information to be separated and a signal from the time domain to the rotation domain can be released, preserving the original rotation harmonics. Therefore, it becomes advisable to eliminate this relationship from the tested signal [41]. Order analysis is a technique mostly used to analyze the signals generated during the operation of machines with rotational or reciprocating components. This approach makes it possible to separate the signal components related to, for example, the rotational speed of the machine, from the remaining signal components, which may contain diagnostic information. In order analysis, successive orders are correlated with the rotational speed (RMP) and harmonics from that speed; therefore, each change of speed gives rise to an order change, and effectively enables a separation from other components of the signal. To some extent, the order analysis makes it possible to assess the proportion of signals generated by the mechanisms of operation of machines and residual processes, often correlated with damage. It may therefore enable the development of methods that track the wear or damage to rotary machines.

Thanks to previous applications of order analysis and many successful applications of this method in technical diagnostics, it is currently possible to find research on the development of these algorithms using more advanced methods, such as higher-order spectrum analysis [42,43]. One of such methods is the Cepstrum analysis. For rotary machines, such as gears, bearings, or engines, Cepstrum analysis is a very useful tool for detecting the components of harmonics or the sidebands commonly found in spectra. This is due to the fact that higher-order spectra consist of higher-order moment spectra, which enables the detection of the non-linear interactions between the frequency components [42]. Paper [44] presents the method of the average instantaneous power spectrum as a time–frequency representation of the selected cyclic components. The averaged instantaneous power spectrum reveals phenomena in the signal that are synchronous with the rotational speed. As a result, the phenomenon of modulating different carrier frequencies occurs. This method allows us to provide additional information on the character of the cyclic component of interest.

Order tracking based on the STFT is an effective method for gear fault detection under time-varying rotational speed without using a tachometer. However, for a rolling element bearing, the signal components related to the rotational speed cannot usually be extracted directly from the TFR [45].

As part of the analysis, the Campbell diagram was also determined. The Campbell diagram, represented as a frequency–speed diagram, has been widely used to predict the possible occurrence of resonances in the design and operation phases of rotating machinery. The Campbell diagram is helpful to design and practice engineers for the assessment of the margin of safe operation in both design and field operation processes [46]. A Campbell diagram plot represents a system’s response spectrum as a function of its oscillation regime. On the ordinate axis, eigenfrequencies in Hz are given, and on the abscissa axis, excitations rotate in rpm. The Campbell diagram enables us to present the maximum values according to the adopted diagram scale (Figure 7—on the left side of the Campbell chart visualization representing the range of values divided into levels), allowing for the specification of the characteristic maxima from the order analysis plot and the determination of their values. An example of the application of the Campbell diagram to diagnose fault cases by analyzing the vibrational characteristics of the harmonic reduction gear is presented in [47].

## 4. Results

This paper is limited to the analysis of vibrations registered on the bearing housing directly after the clutch, as the first kinematic node transmitting the drive through the innovative flexible torsion coupling. The selection of the measuring point results from the advantages in terms of the structure of the structural health monitoring system. The vibrations recorded in this place should reveal the damage patterns of the clutch as soon as possible, or constitute a reference set of data on the technical condition and operating parameters of the clutch.

The signals of vibration accelerations in three orthogonal axes (*x*, *y*, and *z*) recorded during the start-up were analyzed. To fully identify the distribution of the values, the signals are presented as time waveforms, three-dimensional STFT charts, and time–frequency maps.

**Figure 8:**
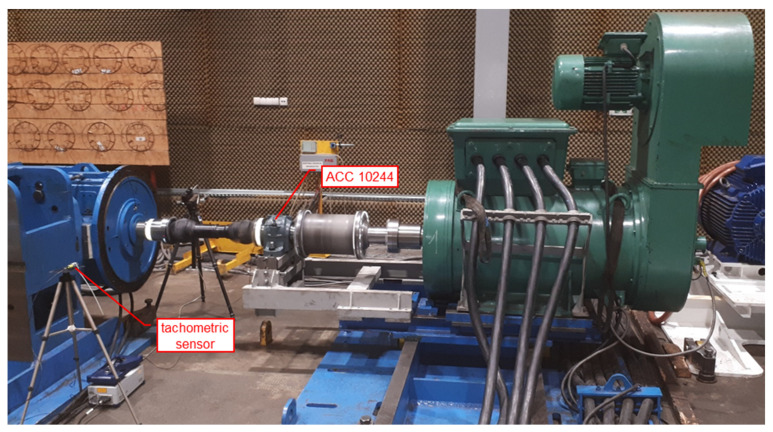
Location of the vibration and tachometric sensors used for the analytical experiment.

Order analysis was performed for the time signal of vibration accelerations recorded by the sensor No. 10244, mounted directly on the bearing housing (Figure 8) directly behind the clutch (looking at the torque transmission from the driving motor). 

**Figure 9:**
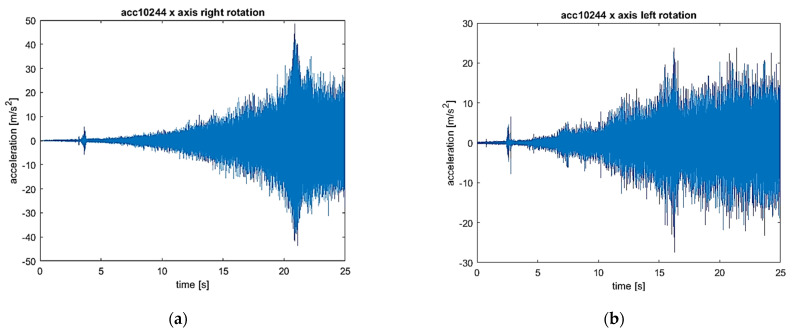

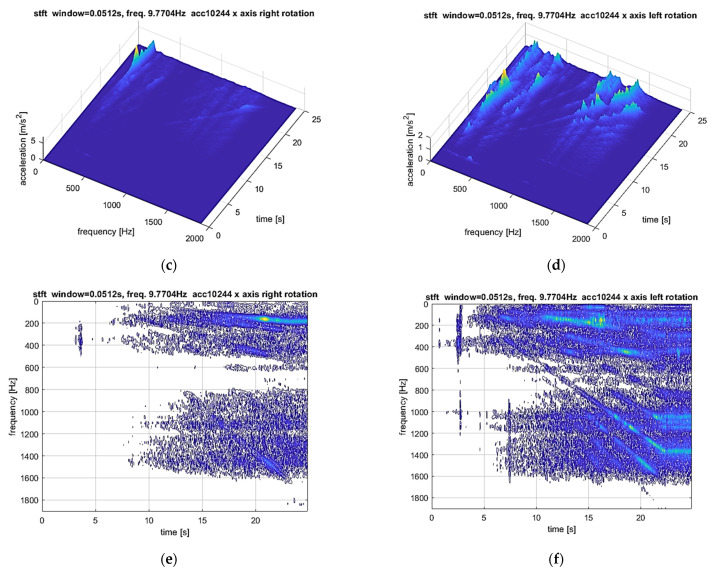
(**a**) Vibration waveform: flexible coupling, direction (axis) *x*; (**b**) vibration waveform: locked coupling, direction (axis) *x*; (**c**) 3-D STFT: flexible coupling, direction (axis) *x*; (**d**) 3-D STFT: locked coupling, direction (axis) *x*; (**e**) map of STFT: flexible coupling, direction (axis) *x*; and (**f**) map of STFT: locked coupling, direction (axis) *x*.

**Figure 10:**
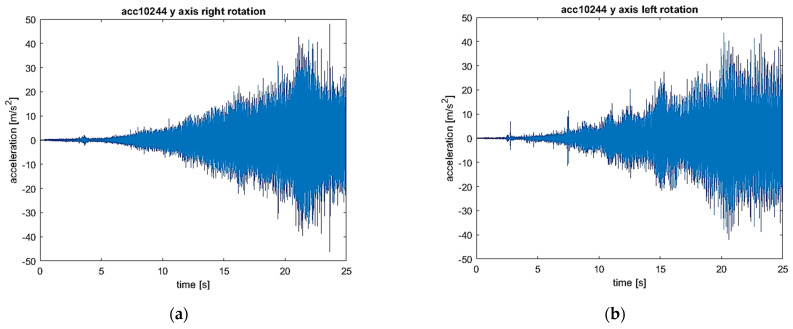

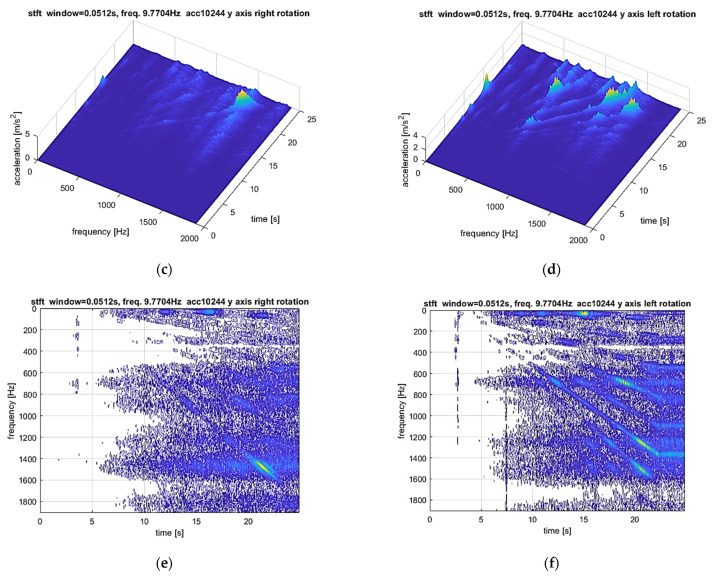
(**a**) Vibration waveform: flexible coupling, direction (axis) *y*; (**b**) vibration waveform: locked coupling, direction (axis) *y*; (**c**) 3-D STFT: flexible coupling, direction (axis) *y*; (**d**) 3-D STFT: locked coupling, direction (axis) *y*; (**e**) map of STFT: flexible coupling, direction (axis) *y*; and (**f**) map of STFT: locked coupling, direction (axis) *y*.

**Figure 11:**
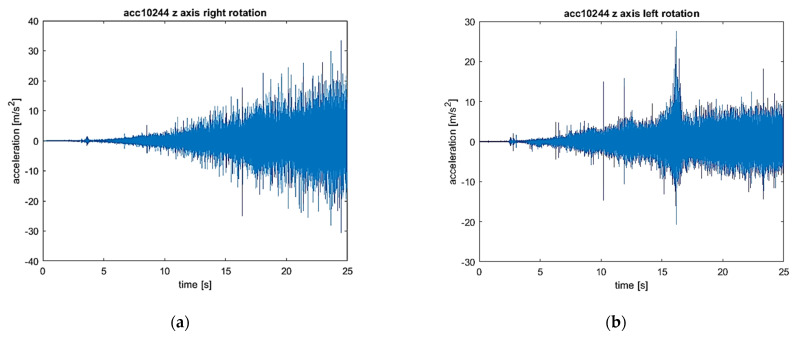

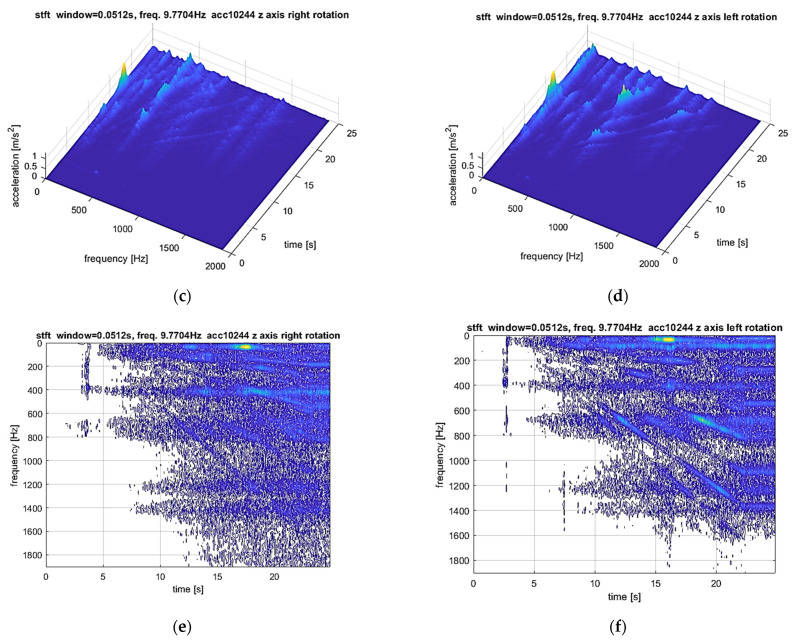
(**a**) Vibration waveform: flexible coupling, direction (axis) *z*; (**b**) vibration waveform: locked coupling, direction (axis) *z*; (**c**) 3-D STFT: flexible coupling, direction (axis) *z*; (**d**) 3-D STFT: locked coupling, direction (axis) *z*; (**e**) map of STFT: flexible coupling, direction (axis) *z*; and (**f**) map of STFT: locked coupling, direction (axis) *z*.

The results set out separately for the vibrations of the *x*, *y*, and *z* axes are presented in Figure 9, Figure 10 and Figure 11. In each figure, the left-hand side (series a) shows the results for the flexible coupling, and the right-hand side (series b) shows the results for the locked coupling.

**Figure 12:**
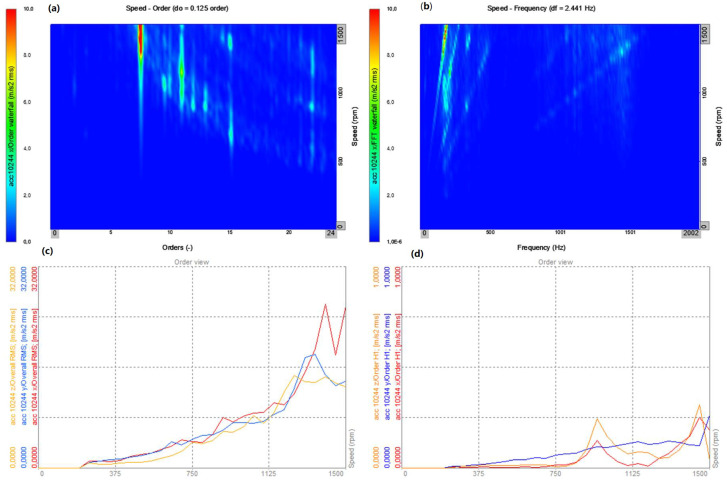
Start-up—flexible coupling (right rotation): (**a**) top view on the plane: order–rotational speed (rpm); (**b**) top view on the plane: frequency–rotational speed (rpm); (**c**) amplitude changes all the harmonics as a function of the rotational speed (rpm) for the three directions (*x*, *y*, *z*); and (**d**) changes in the amplitudes of the first harmonic as a function of rotational speed (rpm) for the three directions (*x*, *y*, *z*).

**Figure 13:**
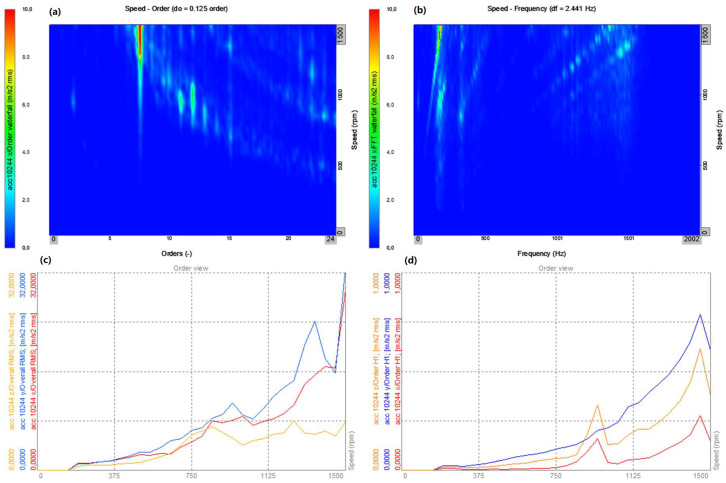
Start-up—locked coupling (left rotation): (**a**) top view on the plane: order–rotational speed (rpm); (**b**) top view on the plane: frequency–rotational speed (rpm); (**c**) amplitude changes all the harmonics as a function of the rotational speed (rpm) for the three directions (*x*, *y*, *z*); and (**d**) changes in the amplitudes of the first harmonic as a function of the rotational speed (rpm) for the three directions (*x*, *y*, *z*).

**Figure 14:**
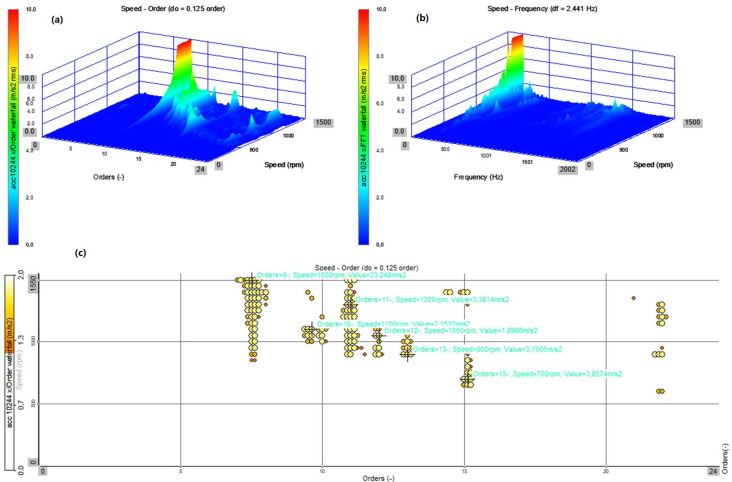
Start-up—flexible coupling (right rotation): (**a**) 3D view on the plane: order–rotational speed (rpm); (**b**) 3D view on the plane: frequency–rotational speed (rpm); and (**c**) Campbell’s diagram representing the range of amplitude values divided into levels and presented as a function of the order and rotational speed (rpm).

**Figure 15:**
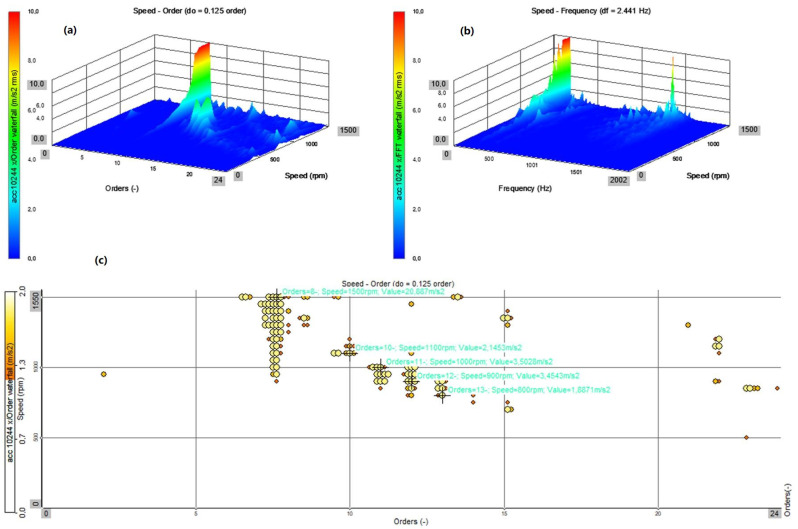
Start-up—locked coupling (left rotation): (**a**) 3D view on the plane: order–rotational speed (rpm); (**b**) 3D view on the plane: frequency–rotational speed (rpm); and (**c**) Campbell’s diagram representing the range of amplitude values divided into levels and presented as a function of the order and rotational speed (rpm).

The presented analyses (Figure 12, Figure 13, Figure 14 and Figure 15) concern the *x* direction, i.e., the vibrations recorded along the axis of the bearing arrangement and the shaft (Figure 8). Changes in the amplitudes of all harmonics (Figure 12c,d and Figure 13c,d) are shown for all three axes (*x*, *y* and *z*). The results for two directions of rotation of the station were compared.

## 5. Discussion

The results obtained from the research on innovative flexible coupling located between the shaft of the drive motor and the support bearing revealed the dynamics of vibrations generated during start-up. This phase of the drive systems operation is characterized by significant non-stationarity, as well as a temporary increase in the dynamic torques caused by the starting torque of the engine. The task of the clutch is to limit the values of these moments by introducing torsional flexibility into the system. Previous solutions use couplings with limited torsional flexibility not exceeding a few degrees, which does not ensure a sufficient reduction in the dynamic impact, which may manifest itself before early failures of bearings or gear teeth. The essence of the research was to determine whether the compliance of the new clutch up to 200 degrees would provide the sufficient damping of dynamic forces.

When comparing the time–frequency representations obtained in the *x* direction (Figure 9a,b) for the sensitive and blocked modes, it is easy to notice that the spectrum obtained for the susceptible variant is characterized by a smaller number of excitation regions of the vibration acceleration values compared to the blocked variant. In Figure 9c, only 1 maximum is visible, located in the region of 200 Hz, which becomes visible at the end of the system start-up. In the case of the blocked variant, there are at least five such areas, and they become visible throughout the entire start-up period. The observed excitations are most likely caused by the amplification of harmonic vibrations in the subresonance ranges of the entire system. A similar nature of dynamic phenomenon is visible in the case of vibrations in the *y* direction (Figure 10a,b). The only noticeable difference is the value of the frequency, at which the excitation is observed for the variant of the flexible coupling (in the analyzed case it is around 1500 Hz). Only by comparing the vibrations in the *z* direction, no significant differences were found in the obtained frequency spectra (Figure 11a,b).

In both cases, for the operation of the system with a flexible coupling (clockwise rotation) and with a locked coupling (left rotation), the time waveforms and time–frequency distributions as well as the order–domain distributions show a significant similarity. The dominant values are for the 8th order at work with the nominal speed of 1480 rpm, and the local maxima for the orders 11, 12, and 13 (Figure 12a–c and Figure 13a–c). For the averaged order runs (overall rms), the local maximum occurs for the speed of 950 rpm, which is also confirmed by the waveforms for the first harmonic as a function of rotational speed (rpm) (Figure 12d and Figure 13d). The similarity of the operation of the coupling in both operating modes results from the lack of torque load during the start-up (run-up of the system), and the flexible operation of the coupling practically overcomes the inertia resistance during the start-up (slight dynamic torsion of the coupling). 

A comparison of the Campbell’s diagram (Figure 14 and Figure 15), which allows for the display of the dominant amplitude values from the order analysis, confirms the dominant values for the order of 7.5 at the nominal speed of 1480 rpm, and the local maxima for orders 11, 12, and 13 at speeds of about 900 rpm. The significant similarity of the generated diagrams confirms that the operation of the coupling in flexible and locked modes in the absence of an external torque load during start-up does not significantly affect the vibration processes during the run-up of the stand.

The differences in the time–frequency representations evidently prove the damping nature of the coupling operation in the susceptible mode. The limited impact of the resonance interactions of the system in the area of the clutch supported by a bearing indicates the advantage of using torsionally flexible couplings on the durability of the drive system components, especially the bearings. At the entrance, the bevel planetary gears installed in the test system have a very sensitive structural node consisting of bevel stage wheels and heavy-loaded rolling bearings. Limiting the momentary moments to load the drive elements can significantly reduce the negative impact of starting. When calculating the load capacity of the gears and bearings, the load spectrum significantly influenced by starts is taken into account.

To verify and improve the credibility of the analysis of the results, the authors also compared their own results with the research of other authors. Paper [48] presents the velocity vibration amplitude of the flexible coupling of a marine propulsion shafting system employing cardan shafts measured at the bearing housing in transverse direction. The results obtained are comparable with the results obtained during our research. The amplitude response functions for the bearing housing vibration velocity depending on the rotor speed for a symmetrical and asymmetrical system and many more numerical experiments are presented in [49]. The research object was a model of rotating machines mounted on active machine foot mounts using vibration mode coupling by asymmetry. Due to the fundamental differences, the comparison of these test results can only be qualitative. The comparative analysis showed the qualitative similarity of the research results. The distribution of the frequency–rotational speed and Campbell’s diagram (Figure 14b and Figure 15b) is compared to the spectrum cascade plots presented in [50]. These results are considered for the lateral responses of lumped mass points under faults as unbalance–misalignment coupling. An interesting result of the vibration signatures of a rotating system with two shafts, four journal bearings, and a flexible disc coupling under angular misalignment are presented in [51]. Two models aiming to describe a metallic disc coupling efforts were tested: a model presented in the PhD thesis of Tuckmantel [52] and a model by Sekhar and Prabhu of particular flexible coupling under angular misalignment [53].

The comparison of the operation of the innovative clutch (coupling) for both modes presented in the present paper clearly shows the advantage of using an additional flexible mechanism of the system connecting the engine with the transmission. The observed effect of minimizing the vibrations of the drive elements cooperating with a highly flexible clutch is confirmed with this type of flexible coupling working in conjunction with low-loaded systems consisting of planetary gears. For example, in the work [54], Filipowicz showed that the values of the analyzed amplitudes of vibration acceleration measured on the planetary gear housing at the start-up system (without the introduced compliance) were significantly higher than the corresponding amplitudes in the case of using flexible coupling. He also found an increase in the amplitude of the vibration acceleration of the gear housing during the steady operation of the drive. Additionally, in the report verifying [55] the properties of innovative flexible couplings made in a construction dedicated to scraper conveyors (these couplings contained the same flexible mechanism as described in this study, only they had different external dimensions) from the tests, it was found that the vibrations of the acceleration values of the clutch housing operating in the compliant compared to a locked clutch. The study also showed an increase in the energy dissipation caused by the increased torsional flexibility of the coupling by means of thermovision measurements. The compliance of the previous results with the results presented in the paper clearly proves the beneficial effect of the use of a flexible mechanism in a mechanical clutch. However, this study made it possible to learn about the changes in the vibroacoustic effects in the time–frequency domains caused by the action of the flexible coupling, and not only for the analysis of the time courses of vibrations (waveforms), as was the case in previous works. It should be emphasized that the vibration damping ability of the clutch is confirmed by two independent methods.

## 6. Conclusions

The tests carried out and the results obtained from the vibration analyses of the drive system equipped with an innovative prototype of a flexible torsion clutch confirm the high information capacity of the signals. This enables various analyses and precise conclusions. It is very important in the stage of prototyping machines, because it enables the validation of design assumptions and the results of the verification of the simulation test [56,57]. Such an approach minimizes the risk of the developed solution being implemented, and enables the performance of improvement activities. In the case of the mining machines, which are largely responsible for the efficiency of mining processes and, more importantly, for the safety of the work, these are fundamental engineering issues [58,59,60].

The comparison of both the recorded vibration acceleration time signals for both cases of the clutch operation, i.e., in the flexible operation mode of the clutch (i.e., the right direction of rotation) and in the locked mode (i.e., the left direction of rotation), allows one to conclude that the clutch operation mode practically does not affect the nature of vibrations in a transient state, which results from the fact that there is no torque load in the drive train. However, the determined STFT spectra allow us to state that, in the frequency band from about 500–1900 Hz, in the case of all 3 analyzed directions of vibration acceleration for a locked clutch, there are higher amplitude components than in the case of the flexible operation of the clutch. The nature of the waveform visible in the graphs (9–11) allows us to conclude that these are changes directly related to the rotational speed as the first harmonic and subsequent harmonics visible as lines not parallel to both axes (time and frequency) in the obtained graphs. The differences for the various operating modes of the clutch may result, inter alia, from a small circumferential play of the clutch.

On the basis of the measurements performed, it can be concluded that the dominant amplitude values are for orders 8 and 11. The comparison of the results obtained for the flexible and locked clutch (i.e., the different directions of rotation: the right and left rotation of the stand) allows one to conclude that there are practically no significant differences in these two cases. This is due to the fact that the clutch does not work, because the start-up took place without load, with the torque coming from the braking motor; hence, the characteristic frequencies are related to the angular gears used in the system operating in the drive transmission system.

The developed research method enables a thorough analysis of the correct operation of the clutch prototype, its effectiveness, reliability, and safety of use in mining conditions.

In future studies, testing will be expanded to verify the results, with comparisons to other independent monitoring systems for different operating scenarios.

## Data Availability

Not applicable.
